# Multiple Sclerosis Heritability Estimation on Sardinian Ascertained Extended Families Using Bayesian Liability Threshold Model

**DOI:** 10.3390/genes14081579

**Published:** 2023-08-02

**Authors:** Andrea Nova, Teresa Fazia, Valeria Saddi, Marialuisa Piras, Luisa Bernardinelli

**Affiliations:** 1 Department of Brain and Behavioral Sciences, University of Pavia, 27100 Pavia, Italy; teresa.fazia01@ateneopv.it (T.F.); luisa.bernardinelli@unipv.it (L.B.); 2 Divisione di Neurologia, Presidio Ospedaliero S. Francesco, ASL Numero 3 Nuoro, 08100 Nuoro, Italy; valeria.saddi@tiscali.it (V.S.); marialpiras@tiscali.it (M.P.)

**Keywords:** heritability, liability threshold model, Bayesian, ascertained families, multiple sclerosis, Sardinia

## Abstract

Heritability studies represent an important tool to investigate the main sources of variability for complex diseases, whose etiology involves both genetics and environmental factors. In this paper, we aimed to estimate multiple sclerosis (MS) narrow-sense heritability (h^2^), on a liability scale, using extended families ascertained from affected probands sampled in the Sardinian province of Nuoro, Italy. We also investigated the sources of MS liability variability among shared environment effects, sex, and categorized year of birth (<1946, ≥1946). The latter can be considered a proxy for different early environmental exposures. To this aim, we implemented a Bayesian liability threshold model to obtain posterior distributions for the parameters of interest adjusting for ascertainment bias. Our analysis highlighted categorized year of birth as the main explanatory factor, explaining ~70% of MS liability variability (median value = 0.69, 95% CI: 0.64, 0.73), while h^2^ resulted near to 0% (median value = 0.03, 95% CI: 0.00, 0.09). By performing a year of birth-stratified analysis, we found a high h^2^ only in individuals born on/after 1946 (median value = 0.82, 95% CI: 0.68, 0.93), meaning that the genetic variability acquired a high explanatory role only when focusing on this subpopulation. Overall, the results obtained highlighted early environmental exposures, in the Sardinian population, as a meaningful factor involved in MS to be further investigated.

## 1. Introduction

Heritability measures the proportion of a trait variability that can be explained by genetic variation [1]. According to the additive model [2,3], the phenotypic variance can be considered as the sum of genetic and environmental effects, and narrow-sense heritability (h^2^) [4] is calculated as the ratio of the additive genetic effects variance on the phenotypic variance. Heritability studies should be considered as the key for discovering potential genetic and environmental causal factors for trait variation characterizing a specific population [1,3,5,6,7]. The results obtained from heritability analyses need to be contextualized relative to the genetic and environmental background of the population under study, highlighting which factor, whether genetic and environmental, has a better explanatory role for the trait variability [1,3,5,7,8].

Quantifying heritability is a major task for complex diseases with uncertain etiology, such as multiple sclerosis (MS) (OMIM 126200), as these are influenced by both genetic and environmental factors [9,10,11]. MS is a chronic autoimmune disease of the central nervous system characterized by inflammation, demyelination, gliosis, and neuronal loss [12,13], and its onset is influenced by both genetic and environmental factors [9,10,11]. Among these, low vitamin D levels, high body mass index, previous Epstein–Barr virus (EBV) infection, and cigarette smoking have been highlighted as strong causal risk factors [14,15,16]. Moreover, several genetic variants were identified as significantly associated with MS susceptibility. Alleles associated with high MS risk were located in the human leukocyte antigen (HLA) complex, while more than 200 non-HLA alleles showed lower MS risk [17,18,19].

MS h^2^ estimates have mainly relied on monozygotic and dizygotic twin pair design [20]; so far, no attempts have been made to estimate this measure in the Sardinian population due to the limited sample size [20,21]. Using extended family-based studies allows overcoming this problem and has the advantage, compared to twin studies, of producing h^2^ estimates less inflated by potential shared environmental effects which could influence individuals raised in a common environment [22,23,24,25].

In this context, different methodologies have been developed to produce unbiased h^2^ estimates for binary traits [26]. However, ascertainment bias arises when using families ascertained from a sampled proband [27,28,29]. To overcome this problem, Kim et al. [29] developed a liability threshold model for binary traits (LTMH) allowing to estimate h^2^, on a liability scale, adjusted from ascertainment bias. However, their expectation–maximization (EM)-based approach presented some limitations, such as lacking a precision measure for h^2^ (i.e., standard error) [30,31], consequent difficulty in calculating confidence intervals [32], computational inefficiency when handling extended families, and convergence issues when including additional variance components in the model, e.g., to adjust for shared environment effects, a feature particularly important when dealing with families and complex diseases.

In this paper, by implementing LTMH methodology in a Bayesian framework using Markov chain Monte Carlo (MCMC) methods to overcome the above described LTMH limitations, we estimated MS h^2^ using 24 Sardinian extended families ascertained from affected probands. Among all, the strength of this sample is represented by the unique characteristics of the founder homogenous Sardinian population and the temporal depth of the available families, which also allows investigating the explanatory role of environmental factors over time, i.e., shared environmental effects, individual environmental effects, sex, and year of birth. The latter can be considered as a proxy for different early environmental exposures due to the post-World War II progressive industrialization and change in socioeconomic factors, dietary habits, lifestyle, and sanitary conditions (“Westernization process”) [33,34,35,36], as well as the malaria eradication program conducted from 1946 to 1950 with the use of insecticide DDT (dichloro-diphenyl-trichloroethane) [37]. These aspects could be linked to the constant MS incidence observed since the 1950s in the Nuoro province [33] and other Sardinian provinces [38]. Different authors have also underlined how a better diagnostic accuracy cannot fully account for this steady increase in MS [33,34,38,39], since the magnitude of this trend has not been observed in any other Italian areas during the same period. Overall, comparing the genetic and environmental impact on MS liability at the population level could have a meaningful impact on the research for MS causal determinants in the population under study [3,6].

## 2. Materials and Methods

### 2.1. Sardinian Families Ascertainment

**Figure 1:**
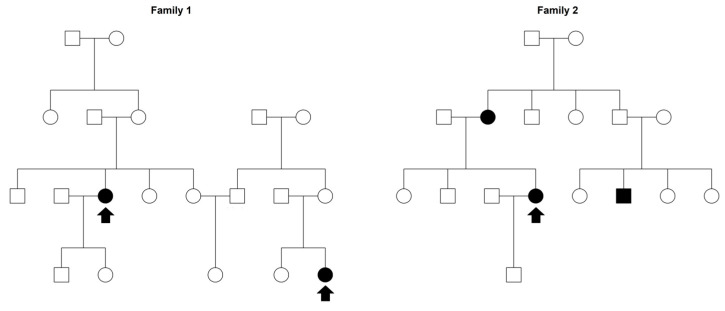
Examples of Sardinian extended families. Multiple sclerosis cases are reported in black, while the arrow denotes a proband.

Our sample was retrieved from a register of MS cases, diagnosed according to Poser’s criteria [40], established in Sardinia’s Nuoro province in 1995. Whenever possible, patients were examined by the neurologists at the Neurology Department of the Nuoro Hospital. Otherwise, clinical records were obtained and reviewed by the previous neurologists. During the examination, the neurologists filled the clinical record of the patient, comprising the MS disease course. From this case register, we sampled 89 MS-affected probands, without any selection in favor of MS patients with a possible family history. Using the genealogical questionnaires filled in by the affected proband and the municipal registries, we were able to reconstruct their genealogical tree. In some cases, MS probands resulted distantly related through a common ancestor, leading to a final sample comprising 24 extended families [19]. Examples of extended families are reported in Figure 1. In our analysis, we included probands’ parents, siblings, spouses, uncles/aunts, first-degree cousins, nieces/nephews, and grandparents, while more distant relatives were excluded to avoid MS misclassifications. Nonaffected relatives included in the final analysis were at least 20 years old at the day of the questionnaire compilation. Thus, a total of 790 subjects were analyzed, comprising 118 MS cases and 672 healthy controls. Descriptive statistics were reported for each family.

### 2.2. Statistical Analysis

#### 2.2.1. Model Specification

To estimate MS h^2^ making use of our ascertained families from a proband, we relied on the LTMH method [29]. Given N individuals clustered in F families, the N observed binary phenotypes (Y) are determined by unobserved continuous liability scores (L) and a fixed threshold (c), which depends on the trait’s prevalence in the population [41]. In our case, MS prevalence was fixed following the work by Montomoli et al. [42], which estimated MS crude prevalence in Nuoro province as 157 per 100,000 inhabitants. We included the following as covariates in the model, to adjust for potential confounding: (i) sex, as the female-to-male MS prevalence ratio in the Nuoro province was reported to be 2:1 [43]; (ii) categorized year of birth (<1946 or ≥1946) as a proxy for the individuals’ different early environmental exposures. L was assumed to be distributed following a multivariate normal distribution, i.e., L~MVN(Xb, Σ), where X denotes a matrix for standardized covariates, i.e., sex and categorized year of birth (YR), b represents the respective vector of fixed effects parameters, i.e., β_SEX_ and β_YR_, and Σ denotes a covariance matrix. We followed the standard polygenic additive model [2,44], assuming null epistatic and gene–environment (G × E) effects, defining Σ as follows (ACE model):

(1) $$\Sigma =h^{2}K+c_{Sibs}^{2}H_{1}+c_{Mother-Offspring}^{2}H_{2}+c_{Father-Offspring}^{2}H_{3}+c_{Spouses}^{2}H_{4}+e^{2}I,$$

where parameters are defined as the proportion of MS liability variability explained by (i) h^2^, additive genetic effects, with K being the kinship matrix multiplied by two, (ii) c^2^_Sibs_, effects due the environment shared between siblings (which also allow to adjust for dominant genetic effects), with H_1_ being the correlation matrix with values equal to 1 between siblings, (iii) c^2^_Mother–Offspring_, effects of environment shared between the mother and the offspring, which may include maternal effects as highlighted in [45,46], with H_2_ being the correlation matrix with values equal to 1 between mother and offspring, (iv) c^2^_Father–Offspring_, effects of environment shared by the father and the offspring, with H_3_ being the correlation matrix with values equal to 1 between father and offspring, (v) c^2^_Spouses_, effects of environment shared between spouses, with H_4_ being the correlation matrix with values equal to 1 between spouses, and (vi) e^2^, individual environmental effects, with I being the respective identity matrix. To avoid identifiability problems [47], e^2^ was derived as the complementary to 1 considering the sum of the other parameters. The proportion of MS liability variance explained by total shared environment effects, i.e., c^2^_Total_, was then defined as the sum of c^2^_Sibs_, c^2^_Mother–Offspring_, c^2^_Father–Offspring_, and c^2^_Spouses_ components. Modeling c^2^_Total_ allows avoiding an inflation in h^2^ due to common environmental influences [23,24,48]. β_SEX_ and β_YR_ allow quantifying the liability increase/decrease and the proportion of MS liability variability jointly explained by both covariates, i.e., τ^2^_βSEX,YR_ = var(Xb) [49]. This latter term can be decomposed, following [50], into

(2) $$\tau_{\beta SEX,YR}^{2}=\tau_{\beta SEX}^{2}+\tau_{\beta YR}^{2}+{2cov}_{\beta_{SEX,YR}},$$

from which we derived the proportion of MS variability marginally explained by (i) sex τ^2^_βSEX_, (ii) categorized year of birth τ^2^_βYR_, and (iii) their covariance component, i.e., 2cov_βSEX,YR_. As described in [49], τ^2^_βSEX,YR_ was considered as part of the total phenotypic variance.

Using the above-specified model, we conducted two separate analyses. In the first, we focused on the whole sample. The explanatory role of G × E effects, between additive genetics and categorized year of birth, was also assessed in a separate model (see Supplementary Section S1 for mathematical details) [51,52]. In the second, we stratified our sample on the basis of the categorized year of birth; the rationale was to evaluate the explanatory influence of genetic and environmental factors on subgroups of individuals with more similar early environmental exposures linked to the year of birth. To better reflect the MS prevalence in these two groups, we relied on the work of Montomoli et al. [42] to set MS prevalence as 103 per 100,000 inhabitants for the individuals born before 1946, and as 176 per 100,000 inhabitants for the individuals born on/after 1946. Only for the analysis on individuals born on/after 1946 did we include the exact year of birth as a continuous covariate in the model to investigate the temporal change in MS liability.

#### 2.2.2. Implementing Bayesian-LTMH

Given the limitations of the EM algorithm implemented in [29], this approach was inefficient to estimate MS h^2^ using our sample since we were dealing with extended families with the aim of including other variance components in the model, e.g., shared environment effects. Therefore, we implemented a Bayesian framework using simulated-based methods as MCMC techniques as it represents an alternative and faster process, compared to maximum likelihood estimation, in the case of complicated statistical models with many unobserved variables [53,54,55,56,57].

In LTMH, the likelihood for the observed phenotypes Y given the unobserved liabilities L and the set of parameters θ = (h^2^, c^2^_Sibs_, c^2^_Mother–Offspring_, c^2^_Father–Offspring_, c^2^_Spouses_, e^2^, β_SEX_, β_YR_, τ^2^β_SEX,YR_, τ^2^β_SEX_, τ^2^_βYR_, 2cov_βSEX,YR_) adjusted from ascertainment bias is defined as follows:

(3) $$p(Y^{NP},L^{NP}|Y^{P},L^{P},\theta )=\frac{p(Y,L|\theta )}{p(Y^{P},L^{P}|\theta )},$$

where P denotes probands, and NP denotes non-probands. The numerator in Equation (3) represents the likelihood function for the complete data, defined by a truncated multivariate normal distribution bounded in the range (a, b) depending on the observed phenotypes Y, i.e., by (−∞, c) if the individual is a control or by (c, +∞) if the individual is a case:

(4) p(Y,L|θ)=L~MVN(Xb,Σ)I(a<L<b).

The denominator in Equation (3) represents the likelihood that the proband is randomly picked from the population and it is necessary to correct for the ascertainment bias. According to the “ascertainment assumption-free” approach [58], this likelihood is defined as

(5) $$p(Y^{P},L^{P}|\theta )=\prod_{i=1}^{F}{(\exp(Y}_{i}^{P}\times \log(\frac{\mu_{i}}{1-\mu_{i}}))\times 1-\mu_{i}),$$

where μ_i_ represents the probability that the liability score for a proband is higher than the threshold c, i.e., μ = P(Y^P^ = 1) = P(L^P^ > c) = 1 − Φ(c − X^P^b). Since multiple distantly related probands could be present within a single family, we considered a single fictitious proband with covariates values equal to the mean of the actual probands’ sex and categorized year of birth within the family.

The conditional likelihood in Equation (3) then served as our sampling distribution for θ parameters. The posterior distributions p(θ|Y, L) could then be characterized using Bayes’ rule as follows:

(6) $$p(\theta |Y,L)\propto p(Y^{NP},L^{NP}|Y^{P},L^{P},\theta )p(\theta )$$

where p(θ) represents the prior distribution specified for the parameters in θ. To the best of our knowledge, there were no previous studies on h^2^ estimation in Sardinian population; therefore, we decided to input noninformative prior distributions for all parameters, i.e., Beta(1,1) for variance components, and N(0,10) for β_SEX_ and β_YR_ parameters. In our analysis, to obtain the sampled parameters’ posterior distributions, we ran four chains with 5000 warmup iterations and 5000 sampling iterations, for a total of 20,000 sampling iterations, and convergence of the four chains to the same posterior distribution was assessed visually using trace plots. Analyses were performed using RStudio, Stan [59,60], and its R interface package CmdStanR [61]. In Supplementary Section S2, we report the results from simulations studies performed across different scenarios to assess the goodness of the Bayesian-LTMH framework.

## 3. Results and Discussion

### 3.1. Sample Description

The analyzed 24 Sardinian families each comprised 7–93 subjects (median = 26) and 1–16 MS cases (median = 3), for a total of 790 subjects: 118 MS cases (15%; 76 females (64%) and 42 males (36%)) and 672 healthy controls (85%). A total of 302 individuals (38%) were born on/after 1946. Descriptive statistics are reported in Table 1.

In Table 2, further details regarding MS cases were reported, including MS course, sex, and age/year of MS onset. The relapse–remitting course (RRMS) was the most represented (49%).

In Table 3, kinship relationships between the MS-related cases within the families were reported; among all these 238 kinship relationships, the distant relationships over the fourth degree were the most represented, i.e., 176 times (74%), while the other kinship relationships (from the first to the fourth) were found in similar proportions.

### 3.2. Bayesian-LTMH Results

We implemented the Bayesian-LTMH, including sex and categorized year of birth as covariates, and no diagnostic problems were encountered. Table 4 reports the results from the first analysis on the whole sample, including the median posterior distributions of the parameters, their standard deviation (SD), and the 95% highest posterior density credibility intervals (HPD CIs).

Categorized year of birth resulted as the strongest explanatory factor for MS liability variability, i.e., τ^2^_βYR_ = 0.69 [95% CI: 0.64, 0.73], meaning that being born before or on/after 1946 explained ~70% of MS liability variability in our Sardinian population. Moreover, compared to individuals born before 1946, individuals born on/after 1946 resulted in a high MS liability increase, i.e., β_YR_ (reference group ≤ 1946) = 3.17 [95% CI: 2.87, 3.48]. This result highlighted year of birth as the major contributor for MS liability variability at the population level, suggesting a crucial role for early environmental exposures which could be related to the so-called “westernization process”, among which different pollution levels, sanitary conditions, and dietary habits other than the sudden lack of *Plasmodium falciparum* immune trigger in the environment consequent to the malaria eradication program. Notably, the latter has been hypothesized to be associated with the increasing Sardinian MS incidence and prevalence observed in the last 50 years [62]. According to this hypothesis, cells of the innate immune system, selected over the centuries to contrast *Plasmodium falciparum* malaria, have kept the tendency to produce abnormal immune responses to new environmental factors even after the disappearance of malaria, consequently leading to an increased autoimmune risk.

Individual and shared environmental factors, not linked to the year of birth, explained ~17% (e^2^ = 0.17 [95% CI: 0.09, 0.23]) and ~8% (c^2^_Total_ = 0.08 [95% CI: 0.02, 0.16]) of MS liability variability, respectively. These could depend on MS risk factors shared between individuals in the same household or specific to the individual, such as past viral infections (e.g., EBV), smoking habits, exposures to pollutants, low vitamin D levels, dietary habits, and childhood/adolescence obesity [15,16,63,64,65,66].

Genetic variability resulted as a poor explanatory factor, i.e., h^2^ = 0.03 [95% CI: 0.00, 0.09]. This result does not imply that genetic variability does not have a causal effect on MS, nor that genetics, in a broader sense, is not involved in determining the disease. Rather, it implies that genetic variability’s contribution in explaining MS liability variability in this specific population is extremely low compared to the other environmental factors. Lastly, sex resulted in a statistically significant increase in MS liability for the “females vs. males” comparison, i.e., β_SEX_ = 0.36 [95% CI: 0.06, 0.68]; however, its explanatory role for MS liability variability was very low compared to the other parameters, i.e., τ^2^_βSEX_ median value = 0.01 [95% CI: 0.00, 0.03].

**Figure 2:**
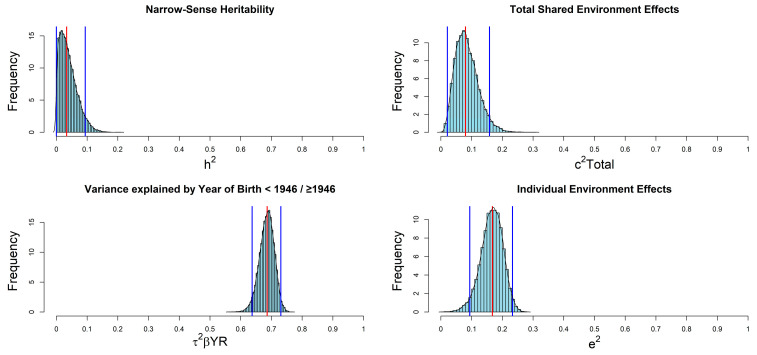
Posterior distributions for parameters included in the Bayesian-LTMH applied to the Sardinian families.

In a separate model, we also included G × E effects variance i.e., h^2^_G×E_, due to interaction between additive genetics effects and categorized year of birth. The estimated h^2^_G×E_ resulted equal to 0.03 [95% CI: 0.00, 0.10], while categorized year of birth remained the main explanatory factor, i.e., τ^2^_βYR_ = 0.69 [95% CI: 0.64, 0.73]. This result does not imply that G × E causal effects were null but indicates that the interaction between early environmental exposures and genetic variants had very little impact on MS variability at a population level, potentially suggesting that these environmental factors may have exerted their effect on MS through other biological mechanisms. The posterior distributions for the parameters are shown in Figure 2, along with median value (in red) and 95% HPD CIs (in blue).

A secondary analysis was conducted stratifying the sample on the basis of the categorized year of birth, thus focusing on individuals with more similar early environmental exposures. The first group, i.e., “<1946”, was composed of 488 subjects: 238 males (49%) and 250 females (51%); 16 MS cases (3%) and 472 healthy controls (97%). The second group, i.e., “≥1946”, was instead composed of 302 subjects: 117 males (39%) and 185 females (61%); 102 MS cases (34%) and 200 healthy controls (66%). Table 5 reports the results from the Bayesian-LTMH model on both groups.

The h^2^ posterior distribution greatly differed between the two groups, i.e., 0.09 [95% CI: 0.00, 0.31] for the “<1946” group and 0.82 [95% CI: 0.68, 0.93] for the “≥1946” group, indicating that genetic variability acquired a high explanatory role for MS liability variability only considering individuals born on/after 1946. For an MS-affected individual born on/after 1946, the high h^2^ value provides a strong likelihood that the genetic variability made a greater contribution compared to environmental factors (specific to “≥1946” group) in producing a deviation from the population MS liability mean [8]. Potential hypotheses to explain the higher value of h^2^ in the second group compared to the first, i.e., (~82% vs. ~9%), could be the following: (i) a decrease in the influence of environmental factors, implying that the genetic variability acquired a higher explanatory role only because the relative explanatory importance was reversed; (ii) an increase in additive genetic effects, implying that the change in environmental factors caused genetic variants to operate differently; (iii) both cases together.

Shared environmental effects and sex resulted as the main explanatory components for the “<1946” group, i.e., c^2^_Total_ = 0.48 [95% CI: 0.21, 0.75] and τ^2^_βSEX_ = 0.31 [95% CI: 0.08, 0.51]; sex resulted in a statistically significant increase in MS liability for “females vs. males” comparison, i.e., β_SEX_ = 1.33 [95% CI: 0.61, 2.03]. Therefore, in this group, specific shared environmental factors (as suggested above), as well as being female, were linked to a higher MS expression at the population level compared to the genetic variability.

Lastly, for the “≥1946” group, we were also able to include the exact year of birth as a covariate, finding a significantly increasing trend in MS liability, i.e., 0.19 [95% CI: 0.01, 0.36] for an increase of 10 years; however, year of birth explained only ~3% of MS liability variability, i.e., τ^2^_βYR_ = 0.03 [95% CI: 0.00, 0.10].

**Figure 3:**
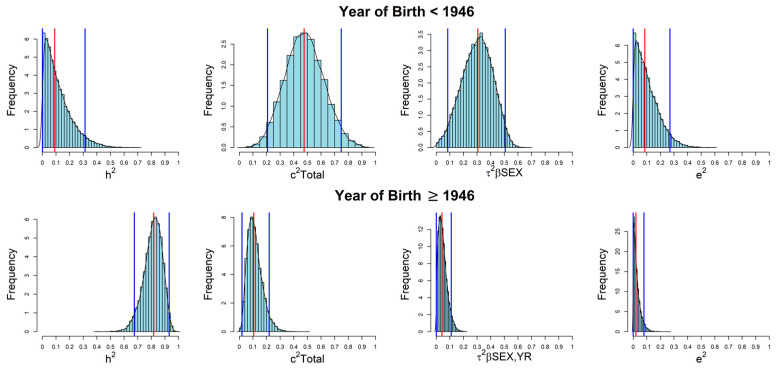
Posterior distributions for parameters included in the Bayesian-LTMH applied to the Sardinian families stratified by year of birth.

In conclusion, the explanatory sources of MS variability largely differed within the two groups given their different early environmental background. The marginal posterior distributions for the parameters are shown in Figure 3 for both groups, along with median values (in red) and 95% HPD CIs (in blue).

Comparing h^2^ estimates between populations, in Sardinian individuals born on or after 1946, it resulted higher (~80%) compared to that obtained using twins from mainland Italy (~50%), Canada (~55%), and the United States (~40%), as well as Finland and France (~25%), while it resulted more similar to h^2^ estimates obtained using twins from the United Kingdom (~75%), as well as Denmark and Sweden (~65%) [20]. These results imply that the genetic variability in the Sardinian population, born on or after 1946, has a better explanatory role for MS liability compared to other populations. This could be due to greater additive genetic effects (e.g., specific genetic variants have a higher risk in the Sardinian environmental background), lower environmental effects (e.g., some of the environmental risk factors present in other population may not be part of the Sardinian environmental background), or both.

It is worth mentioning that our analysis suffered from some limitations. Firstly, available data did not include other potential confounders, even if their effect could have been partially captured in the shared environmental effects. Moreover, the assumed MVN distribution for the underlying liabilities could not be easily checked and, if not respected, could lead to biased estimates [47]. Nevertheless, our Bayesian-LTMH allowed a great advantage to obtain a reasonably precise posterior distribution for MS h^2^ in the Sardinian population using extended families ascertained from a proband [28].

## 4. Conclusions

In line with the latest literature [67], our results pinpoint environmental factors linked to having been born before or on/after 1946 as the leading factors in explaining ~70% of MS liability variability across the 20th century in the Sardinian population. Therefore, further investigations would be crucial to identify these specific early environmental factors involved in the increased MS liability in the Sardinian population. These factors could be researched in the so-called “Westernization process” that took place after World War II, such as different pollution levels, lifestyle, healthcare, and socioeconomic conditions, other than malaria eradication [62]. The remaining variability in MS liability (~30%) resulted mainly explained by environmental factors shared among individuals in the same household or specific to the individual (e.g., low vitamin D levels, obesity, past EBV virus infection, diet, and exposure to pollutants).

Despite the almost null h^2^ obtained analyzing the whole sample, genetic variability remains a highly relevant matter as it acquired the main explanatory role for MS liability variability (~82%) in the individuals born on/after 1946 when performing the stratified analysis based on year of birth. This finding suggests that changes in early environmental factors after 1946 have led to an increased impact of genetic variability on MS at the population level. This could be attributed to either a decline in the impact of environmental effects or a rise in the impact of genetic variant effects on MS risk over time. Therefore, further studies on the Sardinian genetic background could highlight causal biological pathways useful for MS prevention in the current population and for a better understanding of MS etiology.

## Figures and Tables

**Table 1:** Descriptive statistics for the 24 Sardinian families.

Family	Individuals N (%) ^1^	Probands N	Females N (%) ^2^	MS Cases N (%) ^2^
1	65 (8%)	6	37 (57%)	6 (9%)
2	35 (4%)	4	20 (57%)	5 (14%)
3	70 (9%)	7	45 (64%)	9 (13%)
4	66 (8%)	8	37 (56%)	10 (15%)
5	12 (2%)	2	6 (50%)	3 (25%)
6	16 (2%)	2	7 (44%)	2 (13%)
7	43 (5%)	5	24 (56%)	5 (12%)
8	33 (4%)	5	16 (48%)	6 (18%)
9	17 (2%)	2	10 (59%)	2 (12%)
10	20 (3%)	2	13 (65%)	3 (15%)
11	15 (2%)	1	8 (53%)	3 (20%)
12	33 (4%)	5	17 (52%)	6 (18%)
13	17 (2%)	2	11 (65%)	3 (18%)
14	51 (6%)	6	24 (47%)	12 (24%)
15	25 (3%)	3	16 (64%)	3 (12%)
16	44 (6%)	5	24 (55%)	8 (18%)
17	19 (2%)	2	12 (63%)	2 (11%)
18	16 (2%)	2	8 (50%)	2 (13%)
19	22 (3%)	3	13 (59%)	3 (14%)
20	27 (3%)	2	16 (59%)	2 (7%)
21	28 (4%)	1	13 (46%)	2 (7%)
22	16 (2%)	2	7 (44%)	4 (25%)
23	7 (1%)	1	3 (43%)	1 (14%)
24	93 (12%)	11	48 (52%)	16 (17%)
Total	790	89	435 (55%)	118 (15%)

^1^ Percentages refer to the total number of individuals. ^2^ Percentages refer to the number of individuals within the family.

**Table 2:** Descriptive statistics for the 118 multiple sclerosis (MS) cases in the Sardinian families.

MS Course °	N (%)	Females (%)	Age MS Onset Mean (SD)	Year MS Onset Mean (SD)
RRMS	58 (49%)	41 (71%)	28.45 (9.49)	1990 (10.09)
SPMS	27 (23%)	14 (52%)	28.89 (8.87)	1983 (9.64)
PPMS	1 (1%)	1 (100%)	45.00	1995
Unknown	32 (27%)	20 (63%)	N/A	N/A
Total	118	76 (64%)	28.64 (9.06) *	1988 (10.88) *

° RRMS = relapse–remitting MS, SPMS = secondary-progressive MS, PPMS = primary-progressive MS, N/A = not available. * A total of 24 subjects had a missing age of MS onset.

**Table 3:** Kinship relationships between the 118 multiple sclerosis cases.

Kinship Relationship	N (%) *
First degree	20 (8%)
Parent–offspring	9
Mother	6
Father	3
Sibling	13
Second degree	9 (4%)
Uncle/aunt–nephew/niece	8
Grandparent–grandchild	1
Third degree	16 (7%)
Cousins	15
Grand-grandparent–grand-grandchild	1
Fourth degree	17 (7%)
Over the fourth degree	176 (74%)
Total	238

* Percentages refer to the total number of kinship relationships.

**Table 4:** Posterior distributions summary statistics for parameters included in the Bayesian-LTMH applied to the Sardinian families.

Parameter	Median	SD ^1^	HPD 95% CI ^1^
h^2^	0.033	0.028	0.000, 0.094
c^2^_Sibs_	0.033	0.016	0.007, 0.067
c^2^_Mother–Sibs_	0.012	0.012	0.000, 0.039
c^2^_Father–Sibs_	0.013	0.013	0.000, 0.040
c^2^_Spouses_	0.014	0.017	0.000, 0.051
c^2^_Total_	0.080	0.037	0.021, 0.158
e^2^	0.168	0.036	0.094, 0.233
τ^2^_βSEX,YR_	0.712	0.020	0.673, 0.749
τ^2^_βSEX_	0.009	0.008	0.000, 0.027
τ^2^_βYR_	0.686	0.024	0.637, 0.731
2cov°_βSEX,YR_	0.015	0.007	0.003, 0.028
β_SEX(Females vs. Males)_	0.355	0.157	0.057, 0.679
β_YR(≥1946 vs. <1946)_	3.173	0.155	2.869, 3.477

^1^ SD = standard deviation, HPD 95% CI = highest posterior density 95% credibility interval. Proportion of MS liability variability explained by (i) h^2^ = additive genetic effects, (ii) c^2^_Sibs_ = siblings’ shared environment effects, (iii) c^2^_Mother-Sibs_ = shared environment effects between mother and the offspring, (iv) c^2^_Father-Sibs_ = shared environment effects between the father and the offspring, (v) c^2^_Spouses_ = shared environment effects between spouses, (vi) c^2^_Total_ = total shared environment effects, (vii) e^2^ = individual environmental effects, (viii) τ^2^_βSEX,YR_ = sex and year of birth, (ix) τ^2^_βSEX_ = sex, (x) τ^2^_βYR_ = year of birth, and (xi) 2cov°_βSEX,YR_ = covariance between sex and year of birth. β_SEX_ = increase in liability for females compared to males; β_YR_ = increase in liability year of birth on/after 1946 compared to before 1946.

**Table 5:** Posterior distributions summary statistics for parameters included in the Bayesian-LTMH applied to the Sardinian families stratified by year of birth on different environment conditions.

	Year of Birth < 1946			Year of Birth ≥ 1946		
Parameter	Median	SD ^1^	95% HPD CI ^1^	Median	SD ^1^	95% HPD CI ^1^
h^2^	0.090	0.100	0.000, 0.312	0.818	0.068	0.679, 0.937
c^2^_Sibs_	0.223	0.100	0.055, 0.433	0.045	0.030	0.004, 0.109
c^2^_Mother–Sibs_	0.061	0.058	0.000, 0.185	0.013	0.016	0.000, 0.050
c^2^_Father–Sibs_	0.049	0.051	0.000, 0.163	0.014	0.017	0.000, 0.054
c^2^_Spouses_	0.085	0.083	0.000, 0.297	0.019	0.026	0.000, 0.078
c^2^_Total_	0.477	0.142	0.199, 0.750	0.105	0.056	0.019, 0.222
e^2^	0.086	0.083	0.000, 0.265	0.021	0.025	0.000, 0.078
τ^2^_βSEX,YR_	N/A ^1^	N/A ^1^	N/A ^1^	0.042	0.032	0.000, 0.109
τ^2^_βSEX_	0.304	0.112	0.079, 0.506	0.005	0.013	0.000, 0.035
τ^2^_βYR_	N/A ^1^	N/A ^1^	N/A ^1^	0.032	0.030	0.001, 0.095
2cov°_βSEX,YR_	N/A ^1^	N/A ^1^	N/A ^1^	0.000	0.001	−0.001, 0.001
β_SEX(Females vs. Males)_	1.322	0.368	0.586, 2.023	0.104	0.177	−0.246, 0.448
β_YR(10 years increase)_	N/A ^1^	N/A ^1^	N/A ^1^	0.186	0.089	0.012, 0.362

^1^ SD = standard deviation, HPD = highest posterior density credibility interval, N/A = not available. Proportion of MS liability variability explained by (i) h^2^ = additive genetic effects, (ii) c^2^_Sibs_ = siblings’ shared environment effects, (iii) c^2^_Mother–Sibs_ = shared environment effects between mother and the offspring, (iv) c^2^_Father–Sibs_ = shared environment effects between the father and the offspring, (v) c^2^_Spouses_ = shared environment effects between spouses, (vi) c^2^_Total_ = total shared environment effects, (vii) e^2^ = individual environmental effects, (viii) τ^2^_βSEX,YR_ = sex and year of birth, (ix) τ^2^_βSEX_ = sex, (x) τ^2^_βYR_ = year of birth, and (xi) 2cov°_βSEX,YR_ = covariance between sex and year of birth. β_SEX_ = increase in liability for females compared to males; β_YR_ = increase in liability for 10 years increase in year of birth.

## Data Availability

Raw data were uploaded as Supplementary File S2.

## Supplementary Materials

The following supporting information can be downloaded at https://www.mdpi.com/article/10.3390/genes14081579/s1: File S1. R and STAN codes and guidelines for Bayesian-LTMH implementation depending on the number of covariates included in the model; File S2. Dataset of Sardinian families with R and STAN codes to reproduce our analysis; Supplementary Section S1. Mathematical details for the inclusion of G × E effects variance in the Bayesian-LTMH; Supplementary Section S2. Description of the simulation studies conducted to assess the ability of the proposed Bayesian-LTMH to recover the true parameters, along with results reported in a table and in box plots.
