# Supplementary material for: Multiple Sclerosis Heritability Estimation on Sardinian Ascertained Extended Families Using Bayesian Liability Threshold Model
Source: Genes (Basel). 2023 Aug 2;14(8):1579. doi: 10.3390/genes14081579 (PMC10454167; doi:10.3390/genes14081579)
Supplement: Supplementary file 1 [file genes-14-01579-s001.zip › Supplementary Section S1.pdf]

Multiple Sclerosis heritability estimation on Sardinian ascertained extended families using Bayesian liability threshold model

Supplementary Section S1

Modeling the variance of GxE effects

We extended LTMH to investigate the explanatory role of GxE effects between genetic variability and categorized year of birth. This is important to evaluate the validity of the additive principle, which failure would lead to confounded h^2 estimates. This can be investigated and modeled using the approach described in [1], adding in Σ, as defined in Equation (1), the component $h^2_{GxE}K \odot \Upsilon$, where $\odot$ represents the Hadamard product, h^2_{GxE} represents the proportion of MS liability variability explained by GxE effects and Υ a correlation matrix that defines the individuals within the same year of birth group. An estimate for h^2_{GxE} parameter significantly greater than 0 implies that the different impact of additive genetics effects within the different year of birth group have a statistically significant explanatory role for MS liability variability[2]. Consequently, a null h^2_{GxE} parameter must not be interpreted as evidence for the lack of causal GxE effects, but it simply implies a negligible explanatory role for GxE effects.

References

1. Almasy, L.; Towne, B.; Peterson, C.; Blangero, J. Detecting genotype x age interaction. *Genet. Epidemiol.* **2001**, *21 Suppl 1*, doi:10.1002/GEPI.2001.21.S1.S819.
2. Poveda, A.; Chen, Y.; Brändström, A.; Engberg, E.; Hallmans, G.; Johansson, I.; Renström, F.; Kurbasic, A.; Franks, P.W. The heritable basis of gene-environment interactions in cardiometabolic traits. *Diabetologia* **2017**, *60*, 442–452, doi:10.1007/S00125-016-4184-0.
