# Supplementary material for: Multiple Sclerosis Heritability Estimation on Sardinian Ascertained Extended Families Using Bayesian Liability Threshold Model
Source: Genes (Basel). 2023 Aug 2;14(8):1579. doi: 10.3390/genes14081579 (PMC10454167; doi:10.3390/genes14081579)
Supplement: Supplementary file 1 [file genes-14-01579-s001.zip › Supplementary Section S2.pdf]

Multiple Sclerosis heritability estimation on Sardinian ascertained extended families using Bayesian liability threshold model

Supplementary Section S2

Simulation studies

To assess the ability of the proposed Bayesian-LTMH to recover the true parameters we performed simulations under different scenarios. The aim was to evaluate the accuracy and precision of the posterior distribution for the parameters of interest according to the model specification, pedigree structure and trait's prevalence. Therefore, we evaluated posterior distribution uncertainty relative to our prior knowledge, i.e., standard deviation (SD) and lack of bias. To answer these questions, we simulated different scenarios according to i) the pedigree structure, sampling 500 nuclear families or 150 three-generations families from affected probands, ii) trait's prevalence, i.e., 0.05 and 0.005, and iii) the model specification and the effects used to simulate liability scores, which include different combinations of additive genetic effects (A), shared environment effects (C), dominant genetics effects (D), individual environment effects (E), as well as the effect of a single-nucleotide polymorphism (SNP) covariate β_{SNP}. The detailed steps were the following:

1. First, we randomly simulated 150000 nuclear families, with parents having 1,2, 3, or 4 sons/daughters with probabilities 0.2, 0.3, 0.3, 0.2. Sex was always assigned with probability 0.5, and one of the sons/daughters was randomly chosen to represent the proband. In an alternative scenario we randomly simulated 40000 extended pedigrees up to the third generation. The first generation was made of a founders' couple having 2, 3 or 4 sons/daughters with probabilities 0.4, 0.4 and 0.2 which themselves had 1, 2, 3 or 4 sons/daughter with the probabilities 0.2, 0.3, 0.3 and 0.2. Among the second and the third generation, an individual was randomly chosen to represent the proband.
2. Within each family, we simulated liabilities as random draws from a multivariate normal distribution with covariance matrix equal to the sum of the effects specified depending on the scenario and mean equal to 0 or equal to $X\beta_{SNP}$ depending on SNP covariate being included in the model. We evaluated the performance of Bayesian-LTMH fitting: 1) AE model, when liabilities were simulated fixing $h^2 = 0.4$, null c^2, no covariates included; 2) ACE model, modeling c^2_{Sibs}, when liabilities were simulated fixing $h^2 = 0.4$, $c^2_{Sibs} = 0.2$, no covariates included; 3) ACE model, modeling c^2_{Sibs} and $c^2_{Mother\text{-}Offspring}$, when liabilities were simulated fixing $h^2 = 0.4$, $c^2_{Sibs}= 0.2$, $c^2_{Mother\text{-}Offspring}= 0.1$, no covariates included. 4) ACE model as in 2), when liabilities were simulated fixing $h^2 = 0.4$, $c^2_{Sibs} = 0.2$, $d^2=0.2$, no covariates included, to quantify the potential bias in h^2 and c^2 parameters posterior distributions when dominant genetic effects are present but not accounted in the model. 5) ACE model as in 2) but including a SNP as covariate, when liabilities were simulated fixing $h^2 = 0.4$, $c^2_{Sibs} = 0.2$, and SNP effect β_{SNP} explaining 1% of total phenotypic variance, i.e., $h^2_{SNP}= 0.01$. Founder genotypes for each family were generated from a binomial distribution with two trials and the Minor Allele Frequency (MAF) as success probability, which was fixed to 0.2. Non-founder genotypes were consequently obtained following Mendelian transmission. To obtain $h^2_{SNP} = 0.01$, β_{SNP} was fixed to 0.178 following the equation[1]:

$$h^2_{SNP} = \frac{2 * \beta_{SNP}^2 * MAF * (1 - MAF)}{1 + 2 * \beta_{SNP}^2 * MAF * (1 - MAF)} \qquad (Eq. S1)$$

Once liabilities were generated, individuals were considered as cases if their liabilities were larger than a threshold c, which was chosen to maintain the desired cases prevalence. Depending on the scenario, prevalence was fixed as 0.05 or 0.005. Finally, 500 nuclear families and 150 three

generations families were randomly sampled between families with an affected proband, for an expected sample size of ≈ 2400 individuals.

3. Once the ascertained family-based sample was obtained, the Bayesian-LTMH specified according to the scenario was implemented using Hamiltonian Monte Carlo (HMC) to draw samples from the posterior distribution, setting two chains with 1000 warmup iterations and 1000 sampling iterations. Prior distributions were fixed as non-informative Beta distribution, i.e., Beta(1,1), for h^2, c^2_{Sibs} and $c^2_{Mother\text{-}Offspring}$ parameters, and as non-informative normal distribution. i.e., N(0,10), for β_{SNP} parameter.
4. The points 1-3 were repeated 200 times for each scenario. From the obtained parameters' sampled posterior distributions, we calculated different descriptive statistics useful to evaluate the performance of Bayesian-LTMH. The median of the posterior distribution was considered as a point estimate. To evaluate the accuracy of parameters posterior distributions across all 200 simulations, we calculated 1) the median of all point estimates and 2) the bias as the difference from the respective true parameter value. To evaluate the precision, we calculated 3) the SD of all point estimates and 4) the median of all posterior distributions' SDs. Moreover, we calculated 5) the root mean square error (RMSE) as a measure to compare the quality of the posterior distribution, both in terms of accuracy and precision, between scenarios. RMSE is defined as the square root of the mean square difference between the point estimates and the respective true value, i.e., $\sqrt{E[(\widehat{\theta^2} - \theta^2)^2]}$. Finally, we calculated 6) the coverage as the number of times the 95% Highest Posterior Density Credibility Intervals (HPD CIs) contained the true parameter.

Finally, we also compared computational time to fit AE and ACE models using LTHM under the Bayesian framework and under the EM-algorithm approach, considering a sample of 150 three-generations families ascertained from a proband where trait prevalence was equal to 0.005.

Results

Posterior distributions for each parameter were obtained by sampling via the HMC sampler implemented in the program Stan[2]. The performance was evaluated in terms of accuracy, precision, and coverage; **Supplementary Table S1** reports the descriptive statistics for the parameters posterior distributions obtained within each simulated scenario, while **Supplementary Figure S1** reports the corresponding box plots with a red line indicating the true parameter value. No divergences or other diagnostic problems were encountered during HMC sampling. Considering all the scenarios, point estimates for all parameters were generally close to the true value. Therefore, accurate h^2 were obtained in presence of confounders such as shared environmental effects. It can be observed that the RMSE and posterior distribution SD of the estimator across different scenarios showed an increase with i) a lower trait prevalence, or/and ii) increasing the number of variance components in the model, or/and iii) using three-generations families. The latter result can be explained due to decreasing genetic relatedness among distant relatives within a family, such as grandparents-grandchildren or nephews/nieces-uncles/aunts, which led to a lower statistical power compared to the scenario with nuclear families and same sample size. Ascertainment bias was correctly adjusted for β_{SNP} when a SNP covariate was included in the ACE model. A slight downward bias for h^2 parameter was observed when an additional shared environment effect variance component, i.e., $c^2_{Mother\text{-}Offspring}$, was included in the ACE model; this bias was higher when the prevalence of the trait was equal to 0.005 and using three-generations families. When dominance genetic effects variance d^2=0.2 was included in liabilities simulation but not accounted for in the ACE model, the medians of c^2_{Sibs} posterior distributions obtained were, as expected, inflated by a factor corresponding to $0.25d^2$=0.05. However, this adjustment allowed to obtain accurate h^2 posterior distributions, avoiding the inflation from both c^2_{Sibs} and d^2 confounding. Finally, HPD CIs coverage was generally near to 95% in each scenario. Regarding computational efficiency, STAN employed 358.7 seconds to fit an AE model

running one chain with 1000 warmup iterations and 1000 sampling iterations, without requiring multi-threading within-chain parallelization, on a sample of 150 three-generations families. Considering the same sample and number of fixed iterations, STAN employed 401.8 seconds to fit an ACE model including a parameter for c^2. The computational time dropped, respectively, to 124.8 and 140.1 seconds when 10 threads were set for within-chain parallelization. Instead, considering the same sample and the same models, EM-based approach took more than one hour to proceed with a second iteration even after setting 100 threads for parallelization, therefore highlighting the dramatic improvement in speed using the Bayesian framework.

Supplementary Table S1. Descriptive statistics for the sampled posterior distributions obtained fitting Bayesian liability threshold model on the 200 simulated datasets within each different scenario.

Pedigree*	Trait Prevalence	Parameter	Point Estimate Median (SD)**	Bias	SD°	RMSE^	Coverage (95% CI)
**AE model, true h^2 = 0.4**							
Nuclear	0.05	h^2	0.393 (0.046)	-0.007	0.045	0.047	0.94
Three-generations			0.399 (0.061)	-0.001	0.063	0.061	0.95
Nuclear	0.005		0.398 (0.056)	-0.002	0.055	0.057	0.94
Three-generations			0.385 (0.084)	-0.015	0.082	0.087	0.94
**ACE model true h^2 = 0.4, true c^2_{Sibs} = 0.2**							
Nuclear	0.05	h^2	0.400 (0.054)	0.000	0.054	0.054	0.93
		c^2_{Sibs}	0.199 (0.036)	-0.002	0.038	0.036	0.96
Three-generations		h^2	0.399 (0.074)	-0.001	0.073	0.074	0.96
		c^2_{Sibs}	0.197 (0.050)	-0.003	0.051	0.050	0.95
Nuclear	0.005	h^2	0.387 (0.071)	-0.013	0.069	0.072	0.95
		c^2_{Sibs}	0.208 (0.045)	0.008	0.044	0.046	0.97
Three-generations		h^2	0.379 (0.096)	-0.021	0.099	0.098	0.92
		c^2_{Sibs}	0.206 (0.059)	0.006	0.067	0.060	0.97
**ACE model, true h^2 = 0.4, true c^2_{Sibs} = 0.2, true β_{SNP}=0.178, true h^2_{SNP} = 0.01**							
Nuclear	0.05	h^2	0.396 (0.052)	-0.004	0.053	0.052	0.95
		c^2_{Sibs}	0.200 (0.040)	0.000	0.038	0.040	0.95
		β_{SNP}	0.180 (0.062)	0.002	0.067	0.062	0.98
		h^2_{SNP}	0.012 (0.008)	0.002	0.009	0.008	0.98
Three-generations		h^2	0.388 (0.079)	-0.012	0.069	0.080	0.92
		c^2_{Sibs}	0.209 (0.047)	0.009	0.044	0.047	0.94
		β_{SNP}	0.188 (0.091)	0.010	0.096	0.090	0.96
		h^2_{SNP}	0.013 (0.012)	0.003	0.014	0.013	1.00
Nuclear	0.005	h^2	0.393 (0.071)	-0.007	0.073	0.072	0.96
		c^2_{Sibs}	0.202 (0.050)	0.002	0.051	0.050	0.97
		β_{SNP}	0.167 (0.074)	-0.011	0.071	0.074	0.93
		h^2_{SNP}	0.009 (0.009)	-0.001	0.008	0.009	0.93
Three-generations		h^2	0.378 (0.108)	-0.022	0.096	0.110	0.90
		c^2_{Sibs}	0.195 (0.066)	-0.005	0.064	0.066	0.92
		β_{SNP}	0.163 (0.131)	-0.015	0.119	0.130	0.93

		h^2_{SNP}	0.010 (0.017)	0.000	0.014	0.018	0.97
**ACE model, true h^2 = 0.4, true c^2_{Sibs} = 0.2, true $c^2_{Mother\text{-}Offspring}$ = 0.1**							
Nuclear	0.05	h^2	0.382 (0.073)	-0.018	0.073	0.075	0.94
		c^2_{Sibs}	0.202 (0.046)	0.002	0.044	0.047	0.94
		$c^2_{Mother\text{-}Offspring}$	0.102 (0.045)	0.002	0.048	0.046	0.95
Three-generations		h^2	0.385 (0.085)	-0.015	0.087	0.087	0.93
		c^2_{Sibs}	0.214 (0.052)	0.014	0.054	0.054	0.96
		$c^2_{Mother\text{-}Offspring}$	0.102 (0.046)	0.002	0.054	0.046	0.96
Nuclear	0.005	h^2	0.370 (0.091)	-0.030	0.091	0.098	0.93
		c^2_{Sibs}	0.218 (0.053)	0.018	0.053	0.056	0.93
		$c^2_{Mother\text{-}Offspring}$	0.103 (0.052)	0.003	0.056	0.053	0.95
Three-generations		h2	0.353 (0.107)	-0.047	0.112	0.116	0.93
		c^2_{Sibs}	0.218 (0.061)	0.018	0.069	0.065	0.97
		$c^2_{Mother\text{-}Offspring}$	0.104 (0.045)	0.004	0.069	0.046	0.99
**ACE model, true h^2 = 0.4, true c^2_{Sibs} = 0.2 and true d^2 = 0.2**							
Nuclear	0.05	h^2	0.399 (0.056)	0.001	0.054	0.056	0.94
		c^2_{Sibs}+0.25d^2	0.256 (0.038)	0.006	0.037	0.038	0.97
Three-generations		h^2	0.404 (0.075)	0.004	0.073	0.075	0.96
		c^2_{Sibs}+0.25d^2	0.256 (0.052)	0.006	0.051	0.052	0.96
Nuclear	0.005	h^2	0.392 (0.074)	-0.008	0.069	0.074	0.91
		c^2_{Sibs}+0.25d^2	0.253 (0.048)	0.003	0.044	0.048	0.92
Three-generations		h^2	0.376 (0.104)	-0.024	0.099	0.107	0.94
		c^2_{Sibs}+0.25d^2	0.255 (0.067)	0.005	0.065	0.067	0.93

* 500 nuclear families or 150 three-generations families were obtained sampling affected probands

** The point estimate is represented by the median of the posterior distribution

° Median of all posterior distributions' standard deviations

^ Root mean square error

Supplementary Figure S1. Box plots for the sampled posterior distributions obtained fitting Bayesian liability threshold model on the 200 simulated datasets within each different scenario.

1) AE model, true $h^2 = 0.4$

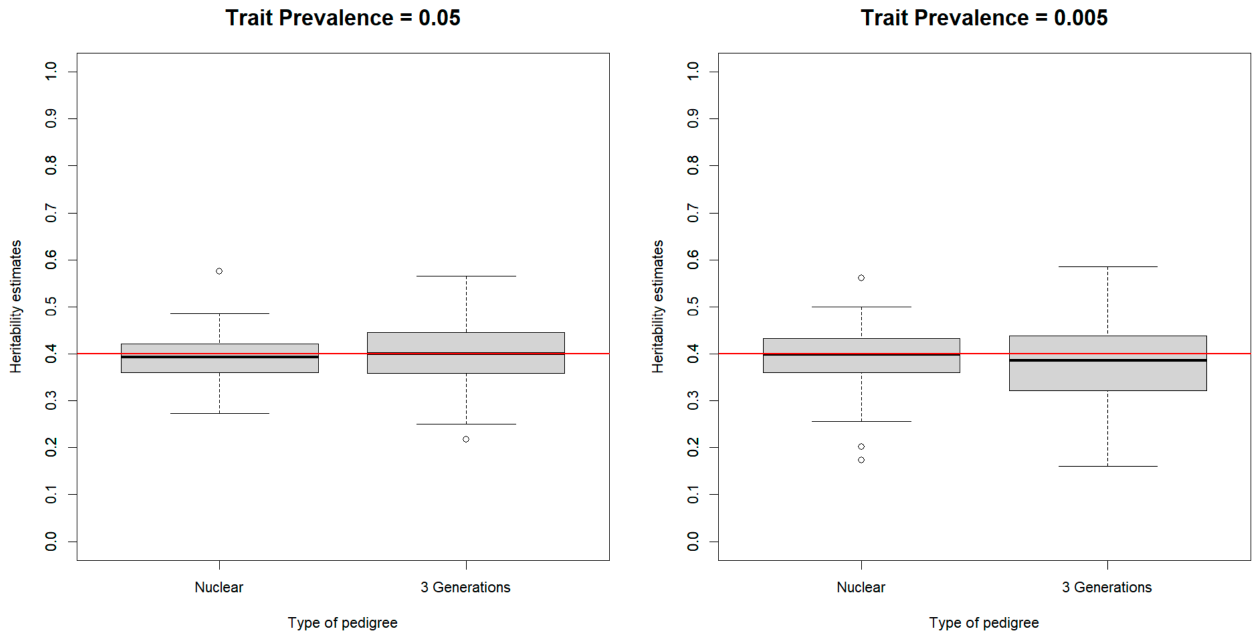

2) ACE model, true $h^2 = 0.4$, true $c^2_{Sibs} = 0.2$

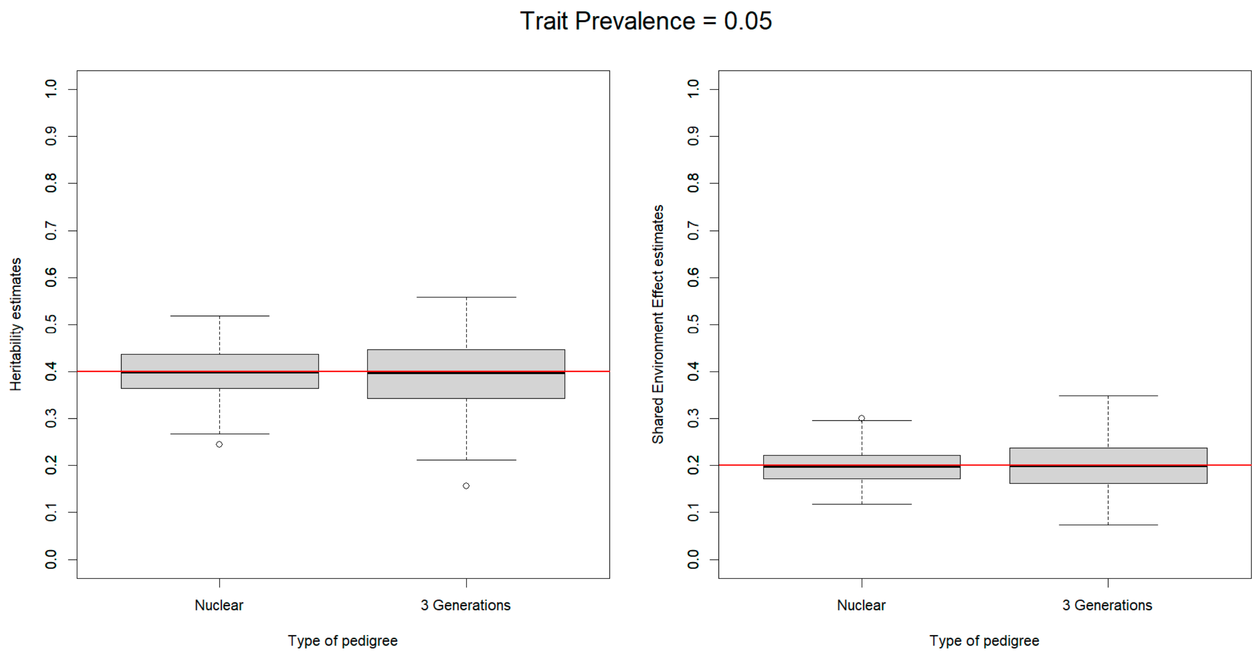

Trait Prevalence = 0.005

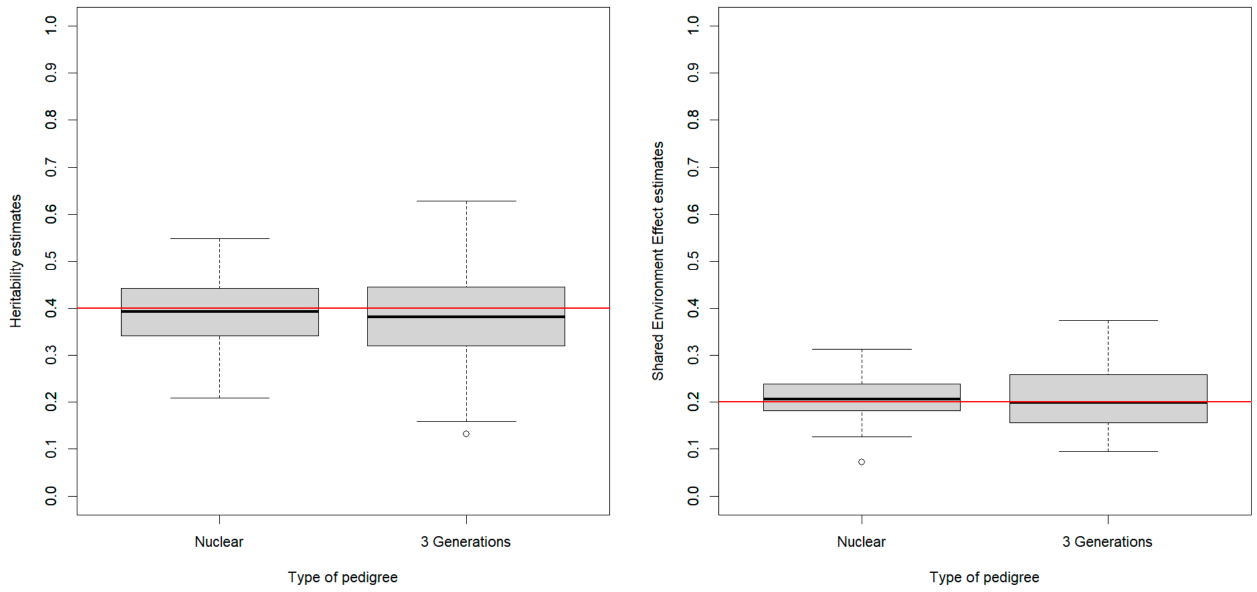

3) ACE model, true $h^2 = 0.4$, true $c^2_{sibs} = 0.2$, true β_{SNP}=0.178, true $h^2_{SNP} = 0.01$

Trait Prevalence = 0.05

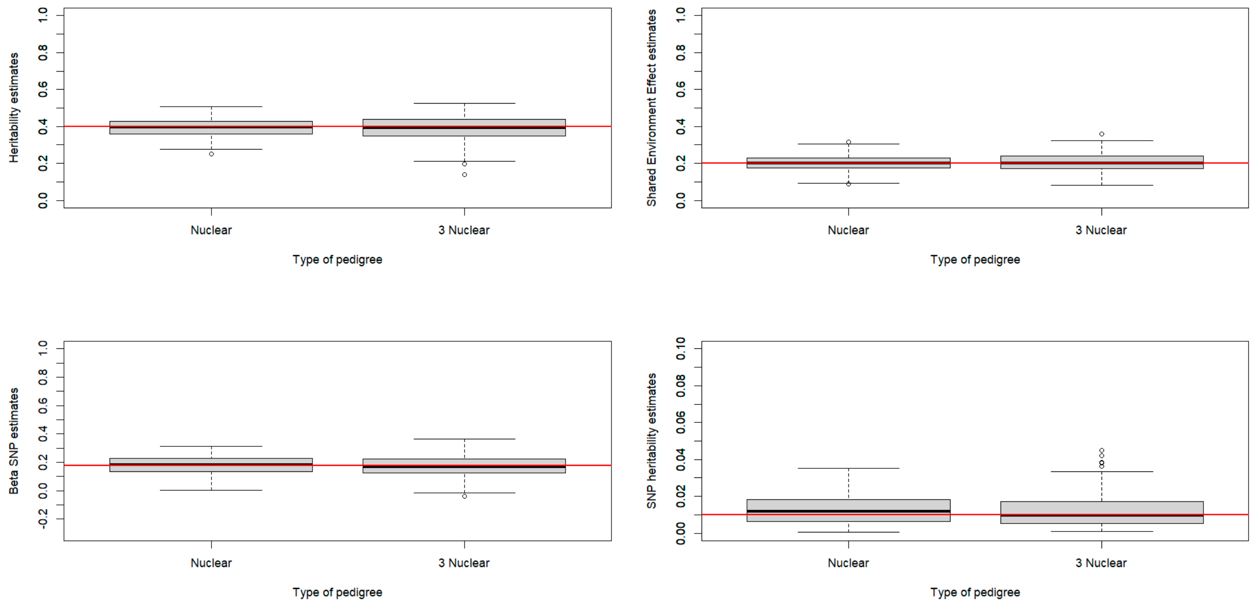

Trait Prevalence = 0.005

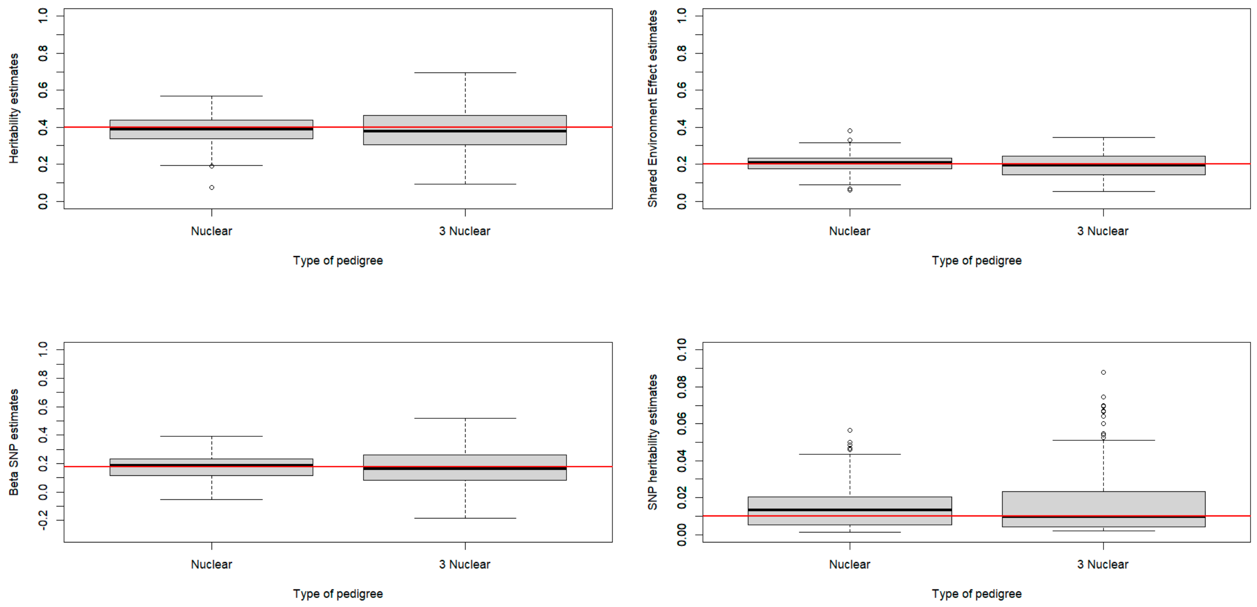

4) ACE model, true $h^2 = 0.4$, true $c^2_{Sibs} = 0.2$, true $c^2_{Mother\text{-}Offspring} = 0.1$

Trait Prevalence = 0.05

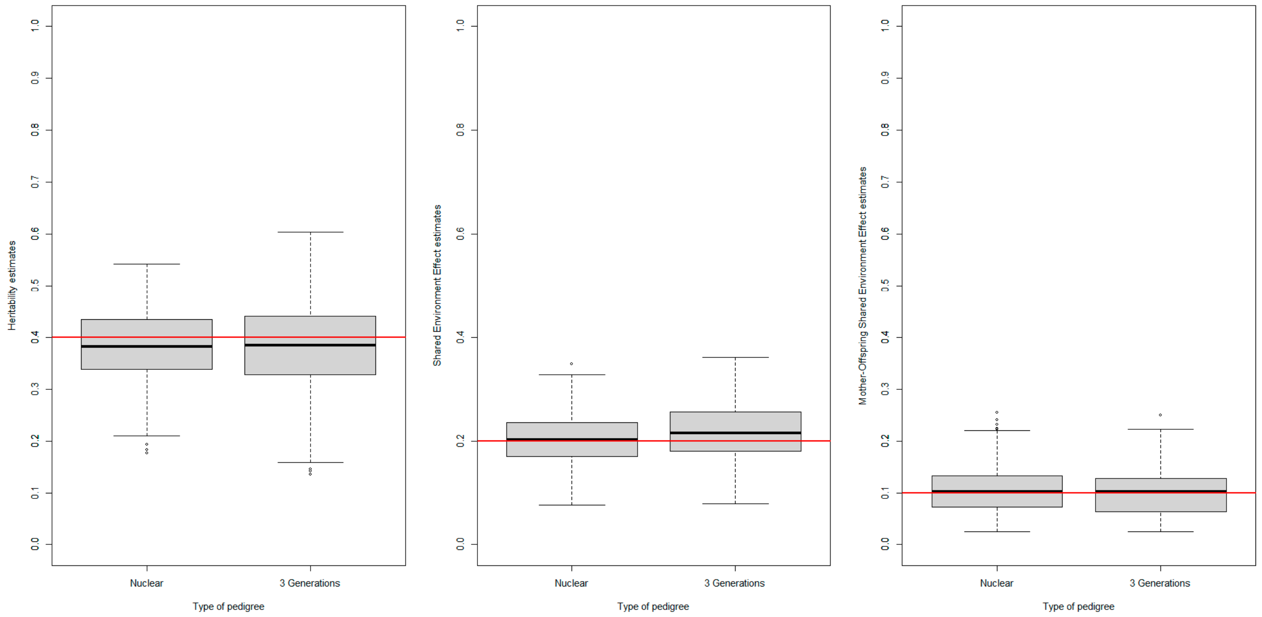

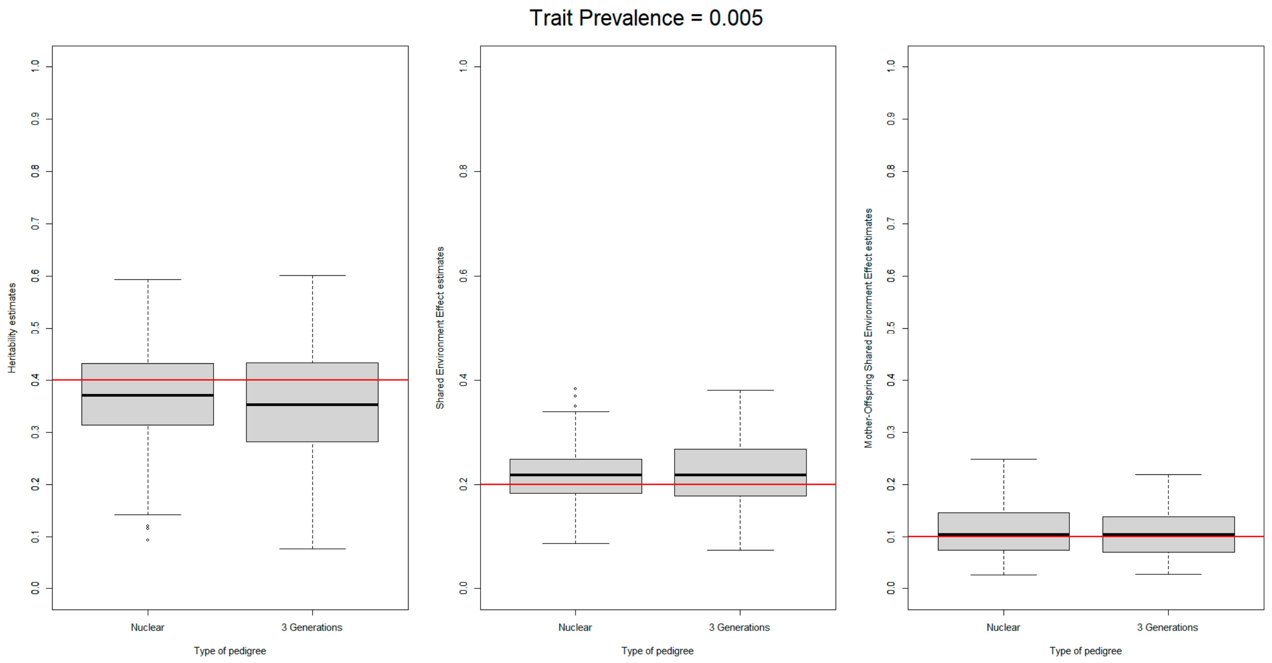

5) ACE model, true $h^2 = 0.4$, true $c^2_{Sibs} = 0.2$ and true $d^2 = 0.2$

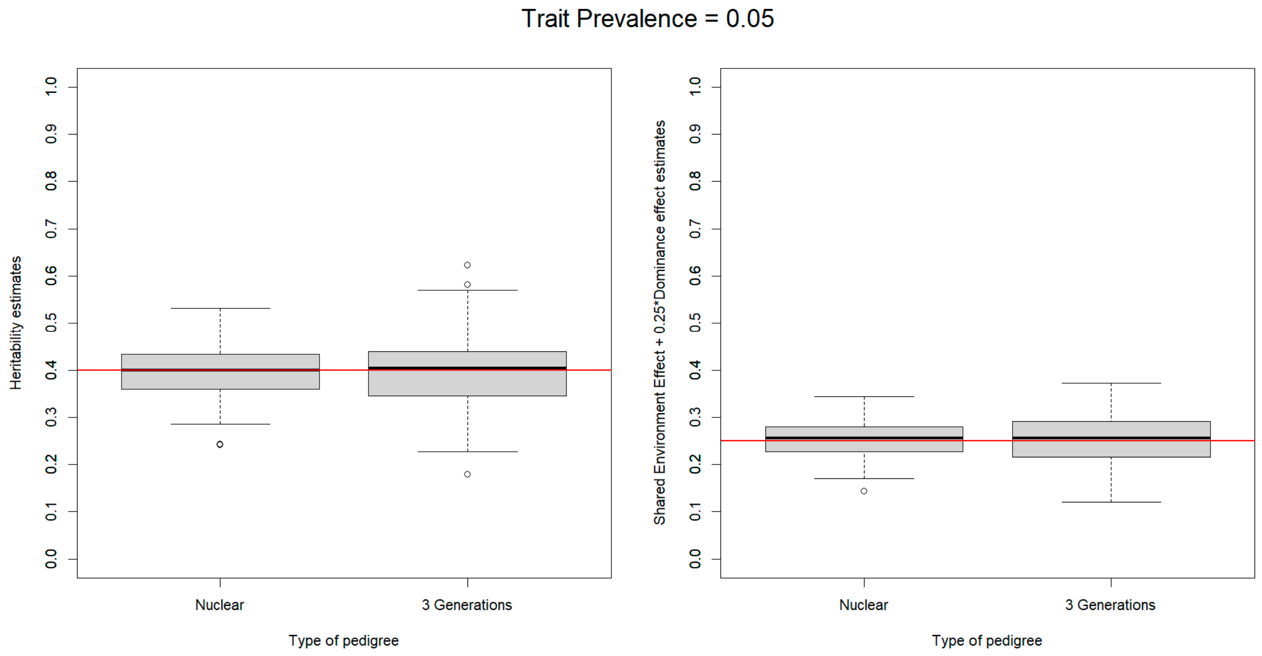

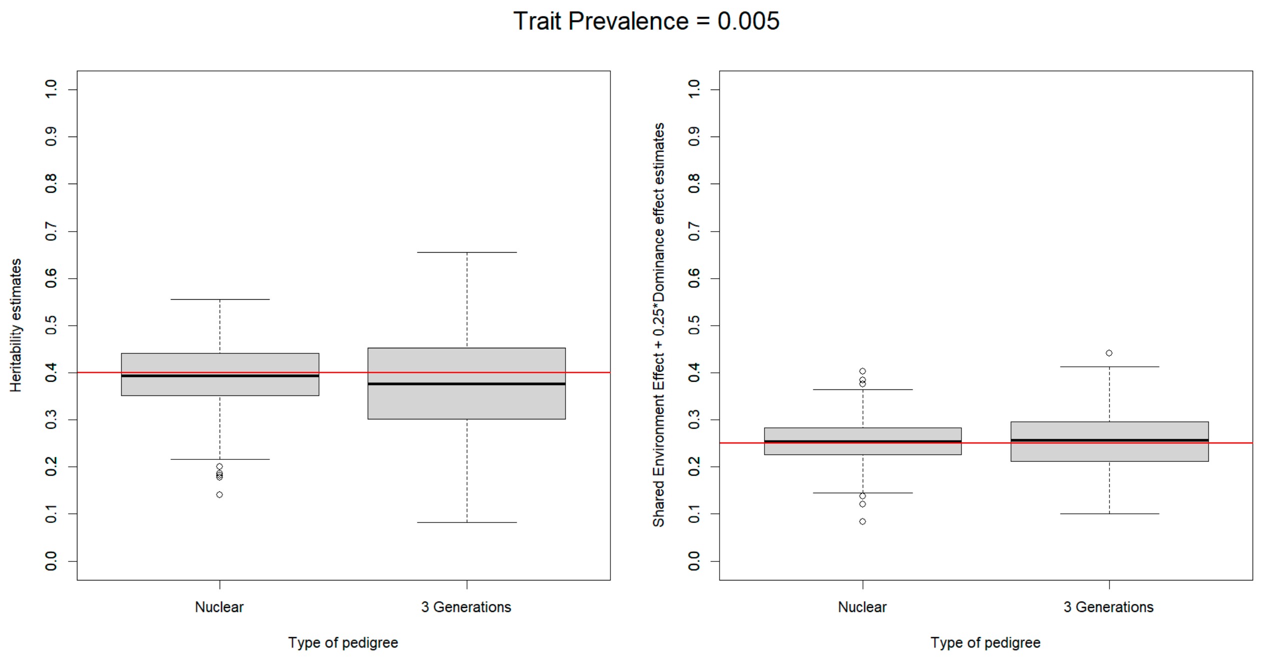

References

1. Kim, W.; Kwak, S.H.; Won, S. Heritability estimation of dichotomous phenotypes using a liability threshold model on ascertained family-based samples. *Genet. Epidemiol.* **2019**, *43*, 761–775, doi:10.1002/GEPI.22244.
2. Carpenter, B.; Gelman, A.; Hoffman, M.D.; Lee, D.; Goodrich, B.; Betancourt, M.; Brubaker, M.A.; Guo, J.; Li, P.; Riddell, A. Stan: A Probabilistic Programming Language. *J. Stat. Softw.* **2017**, *76*, 1–32, doi:10.18637/JSS.V076.I01.
